# Observations on the Botany of Illinois, More Especially in Reference to the Autumnal Flora of the Prairies

**Published:** 1845-03

**Authors:** C. W. Short


### WESTERN JOURNAL

OF

### MEDICINE AND SURGERY.

---

MARCH, 1845.

---

Art. I.—*Observations on the Botany of Illinois, more especially in reference to the Autumnal Flora of the Prairies.* In a letter to Daniel Drake, M.D., &c.

My Dear Sir:

The interest which you have long taken in every thing relating to the Natural History of the Western States, and the desire which you have so frequently expressed to connect the study of Botany with the pursuit of medicine, will, I trust, be a sufficient apology for my addressing these observations to you. Having, moreover, very recently yourself traveled over a part of the country to which they refer, you will be the better enabled to judge of their correctness.

In a tour which I took through the State of Illinois, a few years previous to your own, I had the pleasure of being accompanied by my brother, and one or two other individuals, who took a considerable interest in the objects which mainly prompted my journey; and traveling in a light covered wagon, well prepared for making extensive collections, and vigilantly on the look-out for every object of interest, I may safely say that few such escaped our observation. Our visit to this interesting region was made in the latter part of summer, and extended so late into the fall, that severe frosts had put an end to vegetation before our return; so that my remarks must be considered as referring to the *autumnal Flora* of the Prairies, and may not be applicable to that of the spring, or early summer. We entered the State of Illinois from Terre Haute on the Wabash river, near the line dividing that State from Indiana; thence we traveled in nearly a north-western direction to Peoria, on the Illinois river; through Paris, Urbanna, Bloomington, and Mackinaw: and returning we took a more southern route through Tremont, Springfield, Hillsborough, Maysville, and Lawrenceville to Vincennes; where we recrossed the Wabash, which here forms the boundary between the States of Indiana and Illinois. This trace extending over a distance of nearly 400 miles, led us through the central portion of the State in two different lines, at a considerable distance apart, and gave us an opportunity of seeing and examining the face of the country and its productions under a great variety of aspects.

In a Geographical point of view, the surface of Illinois may be very appropriately, as it is naturally, divided into three districts. First—The heavily timbered tracts which for the most part occupy the southern portion of the State, bordering on the Ohio river, and which, extending into the middle and northern portions, are found in detached bodies surrounded by prairies, and in these situations are called 'Groves.' These groves are, for the most part, contiguous to, and often bounded by water-courses, which have preserved them from the action of fire. Secondly—The open prairies, of from one to twenty miles in diameter, entirely destitute of trees, and indeed of all other woodly plants, except along the margin of water courses which occasionally pervade them. Thirdly—'The Barrens,' or tracts somewhat intermediate between the two former, being sparsely covered with oak trees of several different kinds, and of considerable size, with a dense undergrowth of various shrubs and annual plants. This third region bears a close relationship, both in appearance and productions, to those districts in Kentucky, which are called 'Barrens'—tracts of country which seem to be in a state of transition from more open prairies to densely timbered forests.

The vegetation of these three districts is, of course, essentially different; but apart from the presence or absence of trees, which constitute the grand feature of distinction, the annual and suffruticose plants are widely different, and indeed in many respects entirely dissimilar. It is, however, to the productions of the open prairies that I shall chiefly confine myself in this communication: and even they vary greatly as the surface of the prairie may be high, rolling, rich and dry, or low, flat, wet and clayey.

The first sight of a prairie with which we were greeted was in the neighborhood of Terre Haute, on the eastern side of the Wabash, and consequently in the State of Indiana. In approaching this new and apparently thriving town, from the east, over the national road, the eye is filled with the prospect of an extensive plain entirely destitute of all timber-trees, and stretching to a great distance both above and below the town. Such a view, agreeable at all times, was peculiarly so as it opened suddenly upon us just after emerging from the heavily wooded forest through which we had traveled all day. The Terre Haute prairie, however, has been all reclaimed, or rather, botanically speaking, desecrated by the hand of man, and no portion of it now remains in a state of nature. Corn, grass, small grain, and other cultivated crops now occupy the hundreds of acres, which lately bloomed and blossomed with indigenous productions; and almost the only relics of these to be seen, were occasionally on the road-side, or in fence-corners, a few plants of *Verbena stricta* and *Vernonia corymbosa.*

Twenty miles west of the Wabash at this point, we met with the first prairie in a state of nature; and from this, extending northward to the Lakes, and westward to the Mississippi, they continue, increasing in magnitude, and interrupted only by occasional groves of timber, so as to occupy by far the largest portion of the central, eastern, western, and northern portions of the State of Illinois.

On fairly entering the prairie region, and reaching the centre of one of these immense natural meadows, the view presented to the eye of a novice in such scenery, is one of the most pleasing sort. But beautiful, imposing, and even grand as is this spectacle, I must own, that in a botanical point of view, I was disappointed! The Flora of the prairies—the theme of so much admiration to those who view them with an ordinary eye,—does not, when closely examined by the Botanist, present that deep interest and attraction which he has been led to expect. Its leading feature is rather the unbounded profusion with which a few species occur in certain localities, than the mixed variety of many different species occurring any where. Thus from some elevated position in a large prairie the eye takes in at one glance thousands of acres, literally empurpled with the flowering spikes of several species of *Liatris*, among which the most predominant are *L. spicata*, *L. squarrosa*, *L. scariosa*, *L. cylindracea*, and *L. pycnostachya*. In other situations, where a depressed or flattened surface and clayey soil favor the continuance of moisture, a few species of yellow-flowered *Coreopsis* occur in such profuse abundance as to tinge the entire surface with a golden burnish. The species of this genus more commonly met with in such situations, were *Coreopsis trichosperma*, *C. senifolia*, *C. tripteris*, *C. palmata*, &c., &c. This peculiarity of an aggregation of individuals of one or more species, to something like an exclusive monopoly of certain localities, obtains even in regard to those plants which are the rarest and least frequently met with; for whenever one specimen was found there generally occurred many more in the same immediate neighborhood. The *Dalea alopecuroides*, (Willd.), which I met with but once, was found in that locality in the greatest abundance. The *Satureja hortensis*, which I believe is not regarded as indigenous to North America, was seen once by us in the greatest profusion, and that, too, in a situation the least favorable to the idea of its having been introduced—the centre of a large prairie, where no settlement could have been made. At some places between Peoria and Springfield the road-sides and even the beaten path, were so completely covered over with the little *Boebera chrysanthemoides*, that, trodden under our horses' feet, it exhaled a strong and nauseating odor. In many such localities this noisome weed seems to take the place of the *Anthemis cotula* and *A. arvensis* (Mayweed and Dog-fennel,) in the more settled portions of the Western States. In the neighborhood of Springfield, again, and especially in the out-lots of that town, we found the ground covered, to the exclusion of almost every other vegetation, with a small species of *Ambrosia* (*A. bidentata*) which, at the season in which we saw it, being out of flower, and ripening its dark-colored seed, gave to the common an aspect as dreary as "the bleak and blasted heath where Macbeth met the witches." In illustration of this peculiarity of the Botany of the prairies, I will only further remark that we did not observe the little *Erigeron divaricatum* until we reached Bloomington, in the commons of which town it is extremely abundant; and that it ceases to occur, or is but rarely seen, a few miles south of that.

There are, indeed, comparatively speaking, but few plants, except the grasses, (which are gregarious every where, and are intermixed in greater or less degree and variety among all the other plants of the prairies,) which may be considered as indigenes of the prairie region generally.—Among these we may mention, as occurring most constantly, and under greater diversity of soil and situation than any others, the *Silphium gummiferum*, *Parthenium integrifolium*, *Kuhnia critonia*, *Ceanothus intermedius*, (which here takes the place of *C. Americanus* in the Barrens of Kentucky,) *Prenanthes Illinoensis*, *Eryngium aquaticum*, *Petalostemum violaceum*, *Dracocephalum Virginianum*, *Baptisia leucantha*, several species of *Liatris*, *Coreopsis*, *Aster*,* *Solidago*,† *Rudbeckia*,‡ *Helianthus*,§ *Pycnanthemum*, *Gerardia*, *Pedicularis*, *Gentiana*, &c., &c. Those two beautiful plants, for our knowledge of both of which, I believe, we are indebted to Mr. Nuttall, the *Aster sericeus*, and *Amorpha canescens*, are very generally diffused, but not in the same abundance with many others. Indeed, they constitute an exception to the habit of congregation which obtains among so many of their associates.

As is the case, I believe, with the American Flora throughout the United States, and, indeed, the whole Continent, the autumnal botany of the prairies exhibits a large preponderance of the *Compositæ*. Besides those already mentioned, we may here enumerate, as of frequent occurrence, *Chrysopsis mariana*, *Helenium autumnale*, *Boltonia glastifolia and B. asteroides*, *Bidens frondosa and B. chrysanthemoides*, *Eupatorium serotinum*, *E. aromaticum*, *E. ageratoides*, *E. purpureum*, *E. perfoliatum*, *&c.*, *Cnicus glutinosus*, *C. Virginianus*, *C. muticus*, *C. altissimus*, *&c.*, *Silphium laciniatum*, *S. integrifolium*, *S. terebinthinaceum*, *&c.*, *Prenanthes aspera*, *P. virgata*, *P. racemosa*, *P. serpentaria*, *&c.*, *Vernonia fasciculata*, *V. corymbosa*, and one or two other species.

In a farmer's, or rather a grazier's estimation, the grasses would be regarded as the most valuable of the natural productions of the prairies; and we will next mention some of

---

* *Aster lævis*, *A. Novæ Angliæ*, *A. rigidus*, *A. gracilis*, *A. phlogifolius*, *A. concolor*, *A. azureus*, *A. undulatus*, *A. multiflorus*, *A. oblongifolius*, *A. turbinellus*, *A. carneus*, &c.

† *Solidago rigida*, *S. nemoralis*, *S. graminifolia*, *S. Ridellii*, *S. serotina*, *S. speciosa*, *S. Ohioensis*, *S. neglecta*, &c.

‡ *Rudbeckia purpurea*, *R. laciniata*, *R. hirta*, *R. subtomentosa*, *R. pinnata*, &c.

§ *Helianthus angustifolius*, *H. rigidus*, *H. occidentalis*, *H. grosse-serratus*, *H. tomentosus*, *H. mollis*, *H. pubescens*, *H. microcephalus*, *H. tomentosus*, *H. lætiflorus*, &c.

those which are of most frequent occurrence, omitting all reference to the allied tribe of *Cyperaceæ*, but few of which were observed, in consequence, perhaps, of the late season at which our visit was made. Among the most predominant of the *Gramineæ*, on the rich, dry, and rolling prairies are several species of *Andropogon*, as *A. furcatum*, *A. ciliatum*, *A. mutans*, *A. scoparium*, *&c.*, *Aristida tuberculosa*, *A. stricta*, *A. gracilis*, *&c.*, *Elymus Canadensis*, (var. *glaucifolius*.) *E. Virginicus*, *E. mollis*, *&c.*, *Trichodium laxiflorum*, and *Vilfa heterolepsis*. In flat and marshy situations these give place to various species of *Panicum*, as *P. geniculatum*, *P. agrostoides*, *P. dichotomum*, *P. virgatum*, *P. latifolium*, and the universally diffused *P. crus-galli*, *Leersia Virginica*, and *L. oryzoides*, *Spartina polystachya* and *S. cynosuroides*. All these grasses in their young and tender states are eagerly devoured by cattle: as they become harder and less succulent by age, the coarser are rejected and the more tender are sought for. Among these, I believe, the *Vilfa*, before mentioned, is a general favorite, both for grazing and for hay. All of them, however, are cut promiscuously for this purpose, and when they occur, as frequently they do, in large natural meadows, occupying the ground to the almost entire exclusion of other vegetables, they yield a productive return to the labor of the mower; and when well cured make excellent hay. Our horses, which had never before been accustomed to any other than the cultivated grasses, ate this natural hay with great avidity. The quality of these grasses, both for pasturage and mowing, is much improved by the burning of the prairies during the winter, which, destroying the dead and dry stems, affords a better and earlier bite in the spring, as well as a cleaner swath for the scythe: and by protecting certain portions of the prairie from the action of fire until the spring or early summer, vegetation is then so much retarded by a 'late burn,' as the settlers call it, as to afford good pasturage throughout the latter part of the season.

To this action of the fires, which probably for ages have annually passed over these plains, consuming in their progress all relics of vegetable matter, both woody and herbaceous, is perhaps to be mainly ascribed the color of the soil, which for the most part is literally as black as coal, and in some situations of two or three feet in depth. And to this excess of carbonaceous matter, imparted to the soil of these prairies, is it perhaps to be ascribed that their productions, both in cultivated crops and natural growths, are by no means so rank or luxuriant as one might be led to expect. The Indian corn, though well-eared, was not so tall as I have frequently seen it in Kentucky and Ohio, on lands apparently much inferior in fertility; the different kinds of small grain, though heavily-headed, had a much shorter straw; and many of the natural productions, common to the Illinois prairies and the barrens of Kentucky, were less luxuriant in growth than I have observed them to be in the latter district, though the soil of the barrens has not the same appearance of fertility. This subject deserves particular investigation, and an accurate analysis of the prairie soil might lead to very useful practical deductions. One of our fellow-travelers, a farmer by profession, ascribed the appearance, above mentioned, to a 'sourness' in the soil. But the amount of carbonaceous and alkaline matters resulting from such frequent burnings would rather lead to an opposite conclusion.

Among the œconomical and medicinal plants of the prairies may be mentioned *Gentiana ochroleuca*, the roots of which have somewhat the bitterness of the officinal species, (*G. lutea*, of Europe,) *Prenanthes serpentaria*, several species of *Liatris*, the tuberous roots of which are possessed of acrid pungent qualities, and *Eryngium aquaticum;* all these plants have a considerable reputation, which perhaps is but little deserved, against the bites of poisonous serpents, and hence they are known indifferently by the names of 'snake-root,' 'button snake-root,' rattle-snake's masterpiece,' &c. *Frasera verticillata* is not so frequently seen in the more open prairies as in the thinly-wooded barrens. *Polygala Senega* and *Asclepias tuberosa* are abundant in both these localities. The different species of *Silphium* mentioned, exude from their stems a pearly resinous matter, very similar in appearance and sensible properties to turpentine, and used for the same purposes. The roots of the beautiful *Petalostemum violaceum* have a warm pungent quality, which suggested its employment, among the thousand other articles, in the treatment of cholera, and the plant is now known on the prairies as the 'cholera-weed.' Our two most valuable indigenous bitters *Eupatorium perfoliatum* and *Sabbatia angularis* are abundant, and *Aristolochia serpentaria* is seen occasionally in the groves, where various species of dogwood (*Cornus*) are also of frequent occurrence. Mr. J. A. Lapham, of Wisconsin, informs me that in that territory, the *Amorpha canescens* is called 'lead-plant,' from the circumstance of its growth being considered indicative of the presence of that mineral. If the same sign should hold good in Illinois, the whole of the prairies may one day become a mining region.

Ferns are remarkably rare on the prairies; indeed I do not recollect having met with a single specimen of any species of that extensive tribe in the more open prairies. This may, perhaps, be owing to the absence of that shade and constant moisture in which most of these plants delight. On the skirts of the timbered tracts, several kinds occur, which are usually found in the barrens, as *Pteris aquilina*, *Polypodium dryopteris*, and *P. hexagonopterum;* and in the 'groves' I observed many other species common in the Western States. The same remarks will apply, in a good degree, to the tribe of mosses.

I deem it improper to close these desultory remarks, without giving a catalogue, at least, of other common plants, which presented themselves at different places on our route through the prairies. Some of them may have been already incidentally mentioned, but the most of them occurred under circumstances not calling for particular note or comment. They are given as I find them in my note-book, without any kind of order or arrangement.—

*Verbena stricta,*
*V. hastata,*
*Gerardia purpurea,*
*G. flava,*
*G. erecta,*
*G. auriculata,*
*G. quercifolia,*
*Petalostemum candidum,*
*Desmodium,* } of various species.
*Lespedeza,* }
*Euphorbia corollata,*
*Gaura angustifolia,*
*Typha latifolia,*
*Cassia chamæcrista,*
*C. marilandica,*
*Monarda fistulosa,*
*Leptandra Virginica,*
*Lythrum hyssopifolium,*
*Pedicularis pallida,*
*Gillenia stipulacea,*
*Parnassia palustris,*
*Gentiana rubricaulis,*
*G. quinqueflora,*
*Sium latifolium,*
*Archemora rigida,*
*Artemisia caudata,*
*Polygala verticillata,*
*P. ambigua,*
*P. incarnata,*
*Linum rigidum,*
*Potentilla fruticosa,*
*Psoralea floribunda,*
*Boottia sylvestris,*
*Plantago cordata,*
*P. aristata,*
*Cissus Canadensis,*
*Chelone glabra,*
*Angelica triquinata,*
*Epilobium lincare,*
*Lysimachia revoluta, &c.*

Doubtless many other species came under our observation, but being so common in other parts of the Western country, I omitted to note them.

In relation to the botany of the prairies, I have only to add a few remarks on the shrubs which are found among them; for although in the more open districts of this kind no ligneous or perennial stems are permitted to escape the ravages of the annual fires which sweep over them, yet on the margins of 'sloughs,' and along the courses of the small streams which occasionally meander through them, clumps of bushes and clusters of shrubbery are always to be found. These 'roughs,' as they are called, furnish welcome retreats to grazing cattle, and sometimes to the traveler's horse, from that annoying pest of these regions—the prairie fly.* In these thickets the more common productions are the hazle, (*Corylus Americana.*) three species of sumach, (*Rhus glabrum*, *R. copalinum*, and *R. aromaticum*,) several dwarf kinds of plumb, (*Prunus*,) of which the species were not ascertained, two or three varieties of dogwood, (*Cornus sericea*, *C. asperifolia*, *C. alba*, *&c.*,) several species of undetermined willows, (*Salix.*) Besides these, may be mentioned the *Amorpha fruticosa*, *Zanthoxylum fraxineum*, (prickly ash,) *Prinos verticillata*, *Ilex prinoides*, *Aronia melanocarpa*, *Spiræa tomentosa* and *S. salicifolia*, *Symphorea racemosa*, *Cephalanthus occidentalis*, *Rubus*

---

* I regret that I am not Entomologist enough to give the scientific name of this fly. It is, however, but too well known, both by name and its effects, to all those who have had the misfortune to pass through their haunts during the season of their prevalence, which begins in June, and only ends with the recurrence of hard frosts in the fall. I cannot do better, in this place, than to extract from the note-book of one of my fellow-travelers, his account of this tormenting insect:—

"At length fairly on the prairie, its ocean-like expanse—its multifloral hues, and the strange aspect of everything around us, especially of the far-off head-lands of timber, melting into the horizon with 'the mist of blue' which distance gave them, caused us all for some moments to be mute with rapture and admiration; from which delightful trance we were soon aroused by observing the woeful condition of our panting and stamping horses. In fact every part of them not protected by cloths, was covered by blood-thirsty—blood-sucking flies. These insects, unlike all others of their kind I have ever seen, fall upon their prey without buzzing, circumvolation, or prelude of any sort: they dart with the rapidity of shot from the fowler's gun, and as soon as they have touched the animal on which they alight, seem already bedded in his skin, from which they are not to be dislodged but by main force and violence. So greedy were they, and so intent upon one sole object, that they suffered themselves to be pinched off the horses; and so perfect seemed their blood-sucking apparatus, that hardly had they alighted an instant, when on being brushed off, they appeared already gorged with their sanguine food. Vengeance on our part, and commiseration for our horses, induced us frequently to stop, for the purpose of slaughter; and in a few moments our blood-stained hands, and the heaps of slain in the road, gave evidence that we spared not, and were as merciless as the foe whom we encountered.

*villosus*, (blackberry,) *Ribes rotundifolium*, called Illinois gooseberry, of which the fruit, though spinous, makes a delicious tart; together with various species of wild roses, grape-vines, &c.

Though not properly falling within the compass of this communication, the object of which has been to give some account of the autumnal botany of the prairies, yet before I close it, I will venture to add a few remarks on the forest trees of Illinois. These, in the main, do not differ from the productions of similar districts in the timbered lands of Indiana, Ohio, Kentucky, and Tennessee. In Illinois, the richest groves, interspersed through the prairies, are constituted mainly of the same kind of trees which indicate the best soils generally in the Western States, as black walnut, hickories, hackberry, (*Celtis crassifolia*,) sugar-maple, pawpaw, (*Porcelia triloba*,) &c. The thinner lands are clothed chiefly with oaks of various species, hickories and gums, (*Liquidambar*

---

We thought, too, at first, that we were relieving our horses, *pro hac vice* at least, of their torturing assailants; but a very little observation soon made it manifest that where we killed one, a dozen more keen and insatiable would arrive. So there was no way of stopping with a view to relief—the gauntlet had to be run, and the sooner it was over the better. Indeed, an old prairie traveler afterwards told us, in answer to our wonder, expressed at the naked condition of his horses, that the worse the flies were the more rapidly he drove. Such a course, however, appeared to us inhuman; and our horses, good and true though they were 'as ever looked through a halter,' gave evidences that they were not used to such leeching; and we were truly glad when, nine or ten miles on our road, a resting-place presented itself on the skirts of a forest. It is a most fortunate circumstance that these flies do not infect the woodlands, though immediately adjoining their haunts in the tall, thick herbage of the prairies; and we were told that so disgusted are they with the odor of a stable, they will not pursue an animal that takes shelter in one, however rudely and openly constructed of common round logs, without any *chinking* of its various apertures. From my recollection of Western stables generally, I think this fly evinces a great deal of good sense in avoiding them. It is, indeed, a clean, bright, and beantiful insect, of variegated green-and-golden burnish, about the size of, and not unlike the Spanish blistering fly."—*MSS. Notes on a Tour through Illinois, by* J. Cleves Short.

*styraciflua* and *Nyssa* of two or three species,) whilst the poorest soils, those especially of the 'bushy barrens' and 'oak openings,' are occupied mostly with the different kinds of oak, among which the post-oak, (*Quercus obtusiloba*,) and black-jack, (*Q. ferruginea*,) are most prominent. I am able, indeed, to indicate but two trees which are in any way peculiar to the forests of Illinois; and these are the paccan and catalpa. Of these the paccan (*Carya olivæformis*,) is found abundantly on the southern borders of the State, where about Shawneetown and other points on the Ohio river, it constituted a large portion of the original forest; and from these districts great quantities of the nuts have been exported. They are not considered, however, to be equal, either in size or flavor, to the paccan-nuts of Texas. The other tree—the catalpa, (*Catalpa cordifolia*,) I have the authority of General Harrison for saying, is found occasionally, and of large size, in the alluvions of the Wabash river, where he considered it to be certainly a native; in opposition to the opinion of the Abbé Correa, who thought it more probable that the seeds may have been derived from trees planted by the early French settlers of Vincennes and other posts. I have seen this tree in similar alluvions among the dense forests of Henderson county, Kentucky.

Whilst walking over the prairies adjoining the town of Bloomington, in company with our friend Dr. John F. Henry, who resides there, he pointed out to us an extraordinary phenomenon in connexion with vegetation, and one only visible, I suppose, in a prairie country. It was a semicircular, or rather horse-shoe-shaped line of herbage, distinguishable very plainly from the surrounding and included growths, by its darker or deeper green hue. He said that these circles or segments of circles, usually of fifteen or twenty feet diameter, were frequently to be seen in summer, and that it was generally believed they were occasioned by lightning. He described the thunder-storms of this region as sublimely majestic and terrific. We had no opportunity of witnessing this display of Heaven's artillery, during our journey; but in two or three instances afterwards, I think, we observed this singular appearance of the grass on the prairies, indicating what might, perhaps, without impiety, be called 'the foot-prints of the Deity!'

Very respectfully and truly, I am,

My dear Sir, yours,

C. W. SHORT.

Professor Drake,
Medical Institute of Louisville,

February, 1845.

